# A novel aquaporin-4-associated optic neuritis rat model with severe pathological and functional manifestations

**DOI:** 10.1186/s12974-022-02623-7

**Published:** 2022-10-27

**Authors:** Yuko Morita, Takahide Itokazu, Toru Nakanishi, Shin-ichiro Hiraga, Toshihide Yamashita

**Affiliations:** 1 Department of Molecular Neuroscience, Graduate School of Medicine, Osaka University, Suita, Osaka Japan; 2 Department of Neuro-Medical Science, Graduate School of Medicine, Osaka University, Suita, Osaka Japan; 3 WPI Immunology Frontier Research Center, Osaka, Japan; 4 Graduate School of Frontier Biosciences, Osaka University, Osaka, Japan

**Keywords:** Neuromyelitis optica, Axonal degeneration, Neuroinflammation, Macrophages/microglia activation, Minocycline, Optic neuritis

## Abstract

**Background:**

Optic neuritis (ON) is a common manifestation of aquaporin-4 (AQP4) antibody seropositive neuromyelitis optica (NMO). The extent of tissue damage is frequently severe, often leading to loss of visual function, and there is no curative treatment for this condition. To develop a novel therapeutic strategy, elucidating the underlying pathological mechanism using a clinically relevant experimental ON model is necessary. However, previous ON animal models have only resulted in mild lesions with limited functional impairment. In the present study, we attempted to establish a feasible ON model with severe pathological and functional manifestations using a high-affinity anti-AQP4 antibody. Subsequently, we aimed to address whether our model is suitable for potential drug evaluation by testing the effect of minocycline, a well-known microglia/macrophage inhibitor.

**Methods:**

AQP4-immunoglobulin G (IgG)-related ON in rats was induced by direct injection of a high-affinity anti-AQP4 monoclonal antibody, E5415A. Thereafter, the pathological and functional characterizations were performed, and the therapeutic potential of minocycline was investigated.

**Results:**

We established an experimental ON model that reproduces the histological characteristics of ON in seropositive NMO, such as loss of AQP4/glial fibrillary acidic protein immunoreactivity, immune cell infiltration, and extensive axonal damage. We also observed that our rat model exhibited severe visual dysfunction. The histological analysis showed prominent accumulation of macrophages/activated microglia in the lesion site in the acute phase. Thus, we investigated the possible effect of the pharmacological inhibition of macrophages/microglia activation by minocycline and revealed that it effectively ameliorated axonal damage and functional outcome.

**Conclusions:**

We established an AQP4-IgG-induced ON rat model with severe functional impairments that reproduce the histological characteristics of patients with NMO. Using this model, we revealed that minocycline treatment ameliorates functional and pathological outcomes, highlighting the usefulness of our model for evaluating potential therapeutic drugs for ON in NMO.

**Supplementary Information:**

The online version contains supplementary material available at 10.1186/s12974-022-02623-7.

## Background

Neuromyelitis optica (NMO) is an autoimmune inflammatory disease of the central nervous system (CNS) that mainly affects the optic nerve and spinal cord [1–3]. Most patients with NMO are reported to be positive for antibodies against aquaporin-4 (AQP4), which is expressed in the foot processes of astrocytes [4, 5]. Anti-AQP4 autoantibodies target AQP4 on the surface of astrocytes, causing AQP4 loss and cellular cytotoxicity. This, in turn, induces inflammation, demyelination, and neuronal loss [6]. However, the precise mechanisms underlying neuronal damage remains unclear [7].

Optic neuritis (ON) is a common manifestation of NMO, and compared to multiple sclerosis (MS), the extent of tissue damage is more severe and often leads to loss of visual function [8]. Recently, several new drugs have emerged that can prevent relapse. However, treatment options in the acute phase of NMO attacks are still limited to steroid pulse and plasma exchange therapies [9], which sometimes fail to attenuate the aggravation of visual symptoms [10]. Thus, the development of novel therapeutics that exert ameliorating effects on optic nerve damage caused by NMO attacks is required. Therefore, to elucidate the underlying pathological mechanism and test the effectiveness of potential therapeutic drugs, the establishment of a clinically relevant experimental ON model is necessary.

For this purpose, tremendous research effort has been dedicated, and various kinds of rodent models developed by central or peripheral passive transfer of NMO patient-derived IgG or experimentally generated AQP4-IgG have been reported [11–13]. Nonetheless, previous animal models have resulted in mild optic nerve lesions. In some peripheral passive transfer models, intraperitoneal injection of pathogenic IgG into rats with pre-existing experimental autoimmune encephalomyelitis (EAE) can reproduce the extensive loss of AQP4 with severe tissue damage in the spinal cord and brain. However, optic nerve lesions are very limited. In contrast, the central passive transfer model, which is generated by direct injection of pathogenic IgG into the optic nerve, can reproduce AQP4 loss around the injection site [14–16]. Nevertheless, in previously reported models, the lesion size was small and the severity mild, making it difficult to investigate the therapeutic potential of the tested drugs.

Therefore, in this study, we established a severe ON animal model that reproduces the histological characteristics of ON in seropositive NMO patients, such as the loss of AQP4/glial fibrillary acidic protein (GFAP) immunoreactivity, immune cell infiltration, and extensive axonal damage. For this purpose, we directly injected a high-affinity anti-AQP4 monoclonal antibody, E5415A, which has been shown to induce very severe NMO-like pathology when peripherally injected into rats with pre-existing EAE [17, 18]. Our rat model exhibited severe ON and optic nerve dysfunction with prominent accumulation of macrophages/activated microglia in the lesion site. Therefore, we investigated the possible effect of the pharmacological inhibition of macrophages/microglia activation.

## Materials and methods

### Animals

Female Lewis rats (8–10 weeks, 154–194 g) purchased from Charles River Laboratories were used in all experiments. The rats were housed under a 12-h dark/light cycle with free access to food and water. All experimental procedures were approved by the Institutional Ethics Committee of Osaka University and complied with the Osaka University Medical School Guidelines for the Care and Use of Laboratory Animals.

### Surgical procedures

Rats were randomly divided into two groups: control-IgG (control-IgG-injected rats) and AQP4-IgG (AQP4-IgG-injected rats). An experimental NMO rat model was established by direct injection of AQP4-IgG (E5415A) [19] to the left optic nerve. The surgical procedures were performed according to previous studies [15]. Briefly, the rats were anesthetized with a mixture of vetorphale (10 mg/kg, Meiji Seika Pharma), dormicum (8 mg/kg, Roche), and domitor (0.6 mg/kg, Orion Pharma). Subsequently, they were fixed to a stereotactic frame (SR-5R-HT, Narisige Japan). After exposing the left optic nerve, a small incision was made in the dura using a scalpel to insert the needle. A pulled-glass microcapillary needle attached to a microsyringe (Hamilton) was inserted into the optic nerve 1 mm posterior to the eye globe. Next, 1.5 µL of AQP4-IgG (E5415A) or control-IgG (Mouse IgG2A Isotype Control, R&D Systems) was infused slowly to avoid excessive force. Following the injection procedure, the needle was left in the optic nerve for 2 min to prevent IgG reflux.

**Fig. 1:**
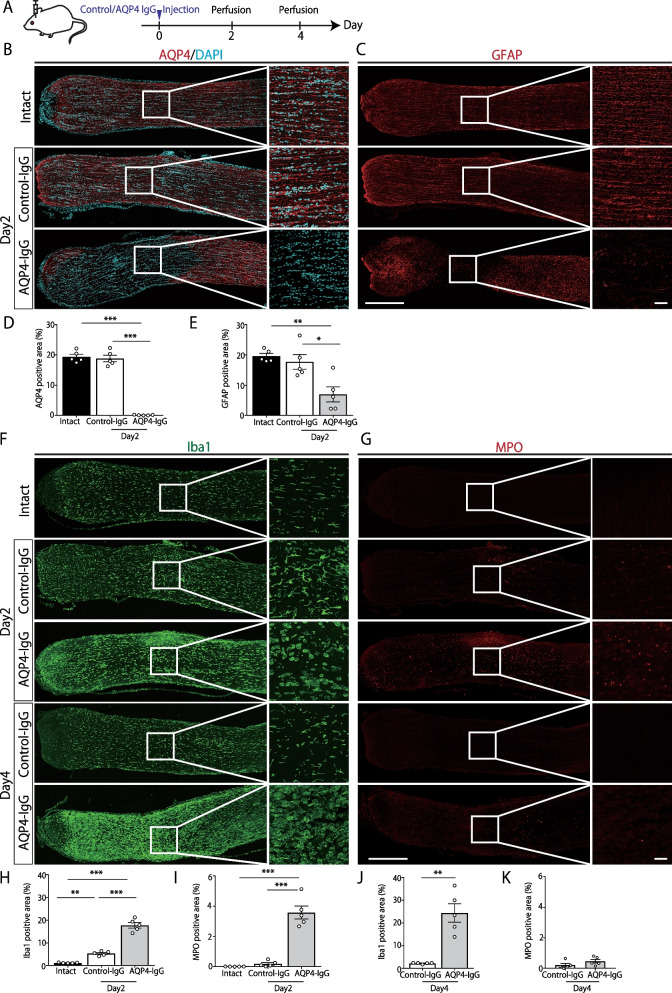
Direct injection of high affinity anti-aquaporin-4 (AQP4) monoclonal antibody into the optic nerve induced neuromyelitis optica (NMO)-like pathology.** A** Experimental time course. Control-immunoglobulin G (IgG) or anti-AQP4 IgG (E5415A) is directly injected into the optic nerve. **B**, **C** Immunohistochemical analysis of Intact, Control/AQP4-IgG-injected rats. Scale bar: 500 μm (low magnification) and 50 μm (high magnification). Longitudinal sections of the optic nerve at 2 days after IgG injection. Representative images of AQP4(red)/Co-immunostaining with DAPI (blue), glial fibrillary acidic protein (GFAP; red). **D**, **E** Quantification of AQP4 and GFAP positive area (%) (Intact: n = 5, Control-IgG: n = 5, AQP4-IgG: n = 5, mean ± SEM, *p < 0.05, **p < 0.01, ***p < 0.001 assessed by one-way analysis of variance (ANOVA) followed by Bonferroni tests). **F**, **G** Immunohistochemical analysis of Intact, Control/AQP4-IgG-injected rats. Scale bar: 500 μm (low magnification) and 50 μm (high magnification). Longitudinal sections of the optic nerve at 2 and 4 days after IgG injection. Representative images of ionizing calcium-binding adaptor protein-1(Iba1: green), and myeloperoxidase (MPO: red). **H**, **I** Quantification of Iba1, MPO positive area (%) (Intact: n = 5, Control-IgG: n = 5, AQP4-IgG: n = 5, mean ± SEM, **p < 0.01, ***p < 0.001 assessed by one-way analysis of variance (ANOVA) followed by Bonferroni test) **J**, **K** Quantification of Iba1, MPO positive area (%) (Control-IgG: n = 5, AQP4-IgG: n = 5, mean ± SEM, **p < 0.01, assessed by Student’s *t* test)

**Fig. 2:**
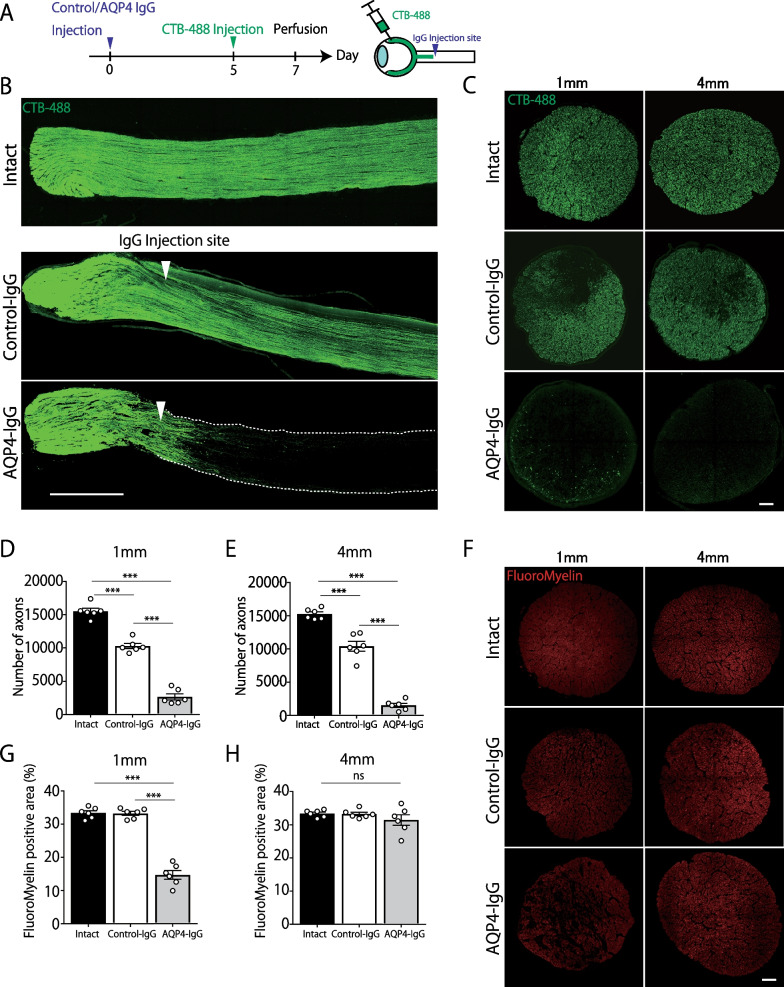
Direct injection of high-affinity anti-aquaporin-4 (AQP4) antibody-induced severe axonal injury. **A** Experimental time course. Cholera toxin subunit b (CTB)–Alexa 488 tracer was injected into the vitreous with a micro-syringe. **B** Representative confocal images of longitudinal optic nerve section. Axons are effectively labeled by CTB. White arrows indicate the injection site. Scale bar: 500 μm. **C** Representative images of CTB-labeled axons in transverse sections at 1 mm and 4 mm posterior to the IgG-injected site. Scale bar: 20 μm. **D**, **E** Quantification of CTB-labeled axons at 1 mm and 4 mm posterior to the IgG-injected site. (Intact: n = 6, Control-IgG: n = 6, AQP4-IgG: n = 6, mean ± SEM, ***p < 0.001 One-way analysis of variance (ANOVA) followed by Bonferroni tests). **F** Representative images of FluoroMyelin in transverse sections at 1 mm and 4 mm posterior to the IgG-injected site. Scale bar: 20 μm. **G**, **H** Quantification of FluoroMyelin-positive area at 1 mm and 4 mm posterior to the IgG-injected site (Intact: n = 6, Control-IgG: n = 6, AQP4-IgG: n = 6, mean ± SEM, ***p < 0.001 One-way analysis of variance (ANOVA) followed by Bonferroni test)

**Fig. 3:**
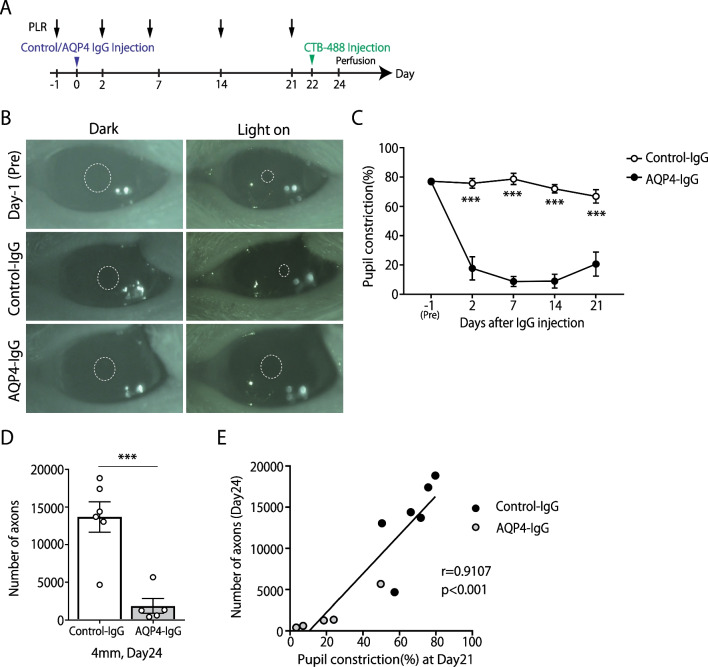
Aquaporin-4 (AQP4)–immunoglobulin G(IgG)-induced optic neuritis (ON) rats exhibit severe visual dysfunction. **A** Experimental time course. Pupillary light reflex (PLR) analysis was conducted on days −1, 2, 7, 14, and 21. **B** Representative images of the pupil recorded in the PLR experiment at before and 7 days after IgG injection. The dotted line indicates the edge of the pupil. **C** Quantification of the pupil constriction (%) (Control-IgG: n = 6, AQP4-IgG: n = 5, mean ± SEM, ***p < 0.001, assessed by two-way analysis of variance (ANOVA) followed by Bonferroni test). **D** Quantification of CTB-labeled axons at 4 mm posterior to the IgG-injected site (Control-IgG: n = 6, AQP4-IgG: n = 5, mean ± SEM, ***p < 0.001 assessed by Student’s *t* test). **E** Spearman's correlation coefficient test was used to assess the correlations between the number of CTB-positive axons (day 24) and constriction rate of Pupillary light reflex (day 21) (Control-IgG: n = 6, AQP4-IgG: n = 5)

**Fig. 4:**
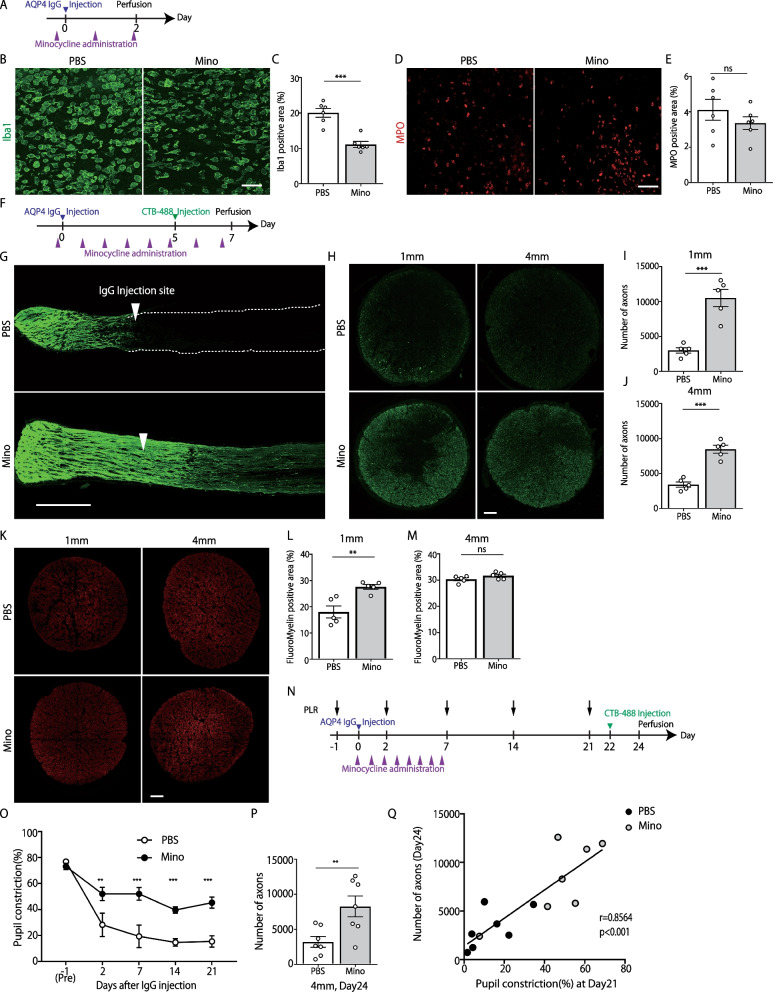
Minocycline ameliorates axonal degeneration and functional deficit in aquaporin-4 (AQP4)–immunoglobulin (IgG)-induced optic neuritis (ON) rats. **A** Experimental time course. **B**, **D** Left: Representative images of Iba1 staining(green). Scale bar: 50 μm. Right: Representative images of MPO staining (red). Scale bar: 50 μm. **C**, **E** Left: Quantification of Iba1-positive area (%) (AQP4-IgG + PBS: n = 6, AQP4-IgG + minocycline: n = 6, mean ± SEM ***p < 0.001, assessed by Student’s *t* test). Right: Quantification of MPO-positive area (%) (AQP4-IgG + PBS: n = 6, AQP4-IgG + minocycline: n = 6, mean ± SEM, ns means no significant difference, assessed by Student’s *t* test). **F** Experimental time course of the analysis on retinal ganglion cell (RGC) axon quantification. **G** Representative confocal images showing CTB-labeled axons in the longitudinal optic nerve section. White arrows indicate the injection site. Scale bar: 500 μm. **H** Representative images of CTB-labeled axons in transverse sections at 1 mm and 4 mm posterior to the IgG-injected site. Scale bar: 20 μm. **I**, **J** Quantification of CTB-labeled axons at 1 mm and 4 mm posterior to the IgG-injected site (AQP4-IgG+PBS: n = 5, AQP4-IgG+minocycline: n = 5, mean ± SEM, ***p < 0.001 assessed by Student’s *t* test). **K** Representative images of FluoroMyelin staining at 1 mm and 4 mm posterior to the IgG-injected site. **L**, **M** Quantification of FluoroMyelin-positive area at 1 mm and 4 mm posterior to the IgG-injected site (AQP4-IgG + PBS: n = 5, AQP4-IgG + minocycline: n = 5, mean ± SEM, **p < 0.01, assessed by Student’s *t* test). **N** Experimental time course of PLR experiment. **O** Quantification of pupillary constriction (%) (AQP4-IgG+PBS: n = 7, AQP4-IgG+minocycline: n = 7, mean ± SEM, **p < 0.001, ***p < 0.001, assessed by two-way ANOVA, followed by Bonferroni test). **P** Quantification of CTB-labeled axons at 4 mm posterior to the IgG-injected site. (AQP4-IgG+PBS: n = 7, AQP4-IgG+minocycline: n = 7, mean ± SEM, **p < 0.01 assessed by Student’s *t* test). **Q** Spearman's correlation coefficient test was used to assess the correlations between the number of CTB-positive axons (day 24) and constriction rate of Pupillary light reflex (day 21) (AQP4-IgG+PBS: n = 7, AQP4-IgG+minocycline: n = 7)

One hundred twenty-four rats were used in this study. ON model establishment: 43 rats for immunohistochemical analysis (Fig. 1, 25 rats, day 2: AQP4-IgG n = 5, Control-IgG n = 5, Intact n = 5, day 4: AQP4-IgG n = 5, Control-IgG n = 5, Additional file 1: Fig. S1, 18 rats), 27 rats for axonal tracing (Fig. 2, coronal section for quantification: AQP4-IgG n = 6, Control-IgG n = 6, Intact n = 6, and sagittal section: n = 9), and 12 rats for behavioral testing (Fig. 3, AQP4-IgG n = 6, Control-IgG n = 6, one rat in AQP4-IgG group was excluded from the analysis because of the wound in the ocular surface) were used. Minocycline treatment (Fig. 4): 12 rats for immunohistochemical analysis, 16 rats for axonal tracing, and 14 rats for behavioral testing were used.

### Minocycline treatment

ON rats were randomly divided into two groups: minocycline (45 mg/kg, M9511, Sigma-Aldrich)-treated group and phosphate-buffered saline (PBS) group. Minocycline/vehicle was administered by intraperitoneal injection 1 h before AQP4-IgG injection and then once daily for 7 days [20].

### Intravitreal cholera toxin subunit B injection

The optic nerve axon was labeled with Alexa Fluor 488-conjugated cholera toxin subunit tracer (CTB-488 Invitrogen) 2 days before the animals were perfused. Tracer injection procedures were performed as in previous studies [21]. Rats were anesthetized and fixed to a stereotactic frame, and a CTB-488 tracer (5 µL; 1 mg/mL in PBS) was infused into the vitreous with a micro-syringe. The needle was left in the vitreous for 2 min to prevent the reflux of the CTB-488. For the quantification of axons at 7 days (Figs. 2A–E, 4F–J), intravitreal CTB injection was performed at 5 days after E5415A/Control-IgG injection, then animals were perfused at 7 days to collect optic nerves. For the correlation analysis between axon number and pupillary light reflex (Figs. 3A–E, 4N–Q), behavioral tests were conducted until 21 days after E5415A/Control-IgG injection. Subsequently, rats received intravitreal CTB injection at 22 days, and they were perfused at 24 days for histological analysis.

### Histological analysis

At 2, 4, 7, and 24 days after the direct injection of IgG, rats were deeply anesthetized and intracardially perfused with 4% paraformaldehyde (PFA) in 0.1 M phosphate buffer. Optic nerves were removed and post-fixed in 4% PFA at 4 °C overnight. After several washes with PBS, the samples were transferred to a 30% sucrose solution. The samples were then embedded in Tissue-Tek O.C.T. Compound (Sakura), cut into 18-µm-thick sagittal and 14-µm-thick coronal sections using a cryostat (Leica Microsystems), and placed on MAS-coated glass slides (Matsunami). For immunohistochemistry (IHC), the sections were incubated in 3% donkey serum in PBS with 0.1% Triton-X for 1 h at room temperature (RT). Then, they were incubated with the following primary antibodies in 3% donkey serum overnight at 4 °C: anti-AQP4 rabbit antibody (1:500; 59678S, Cell Signaling), anti-GFAP chicken antibody (1:1000; ab4674, Abcam), anti-ionizing calcium-binding adaptor protein-1(Iba1) goat antibody (1:500; ab5076, Abcam), and anti-myeloperoxidase (MPO) rabbit antibody (1:100; PA5-16672, Thermo). The sections were washed three times with PBS, followed by incubation with the following secondary antibodies for 1 h at (RT) in the dark: donkey–anti-rabbit IgG Alexa 647 (1:500; A31573, Invitrogen), goat anti-chicken IgG Alexa 568 (1:500; A11041, Invitrogen), donkey–anti-goat IgG Alexa 488 (1:500; A11055, Invitrogen), and donkey anti-rabbit IgG Alexa 568 (1:500; A10042, Invitrogen). For myelin staining, FluoroMyelin™ Red (1:300; F34652, Invitrogen, 30 min at RT) was used. Cell nuclei were stained with 4′,6-diamidino-2-phenylindole (DAPI) (1:1000; D523, Dojindo). The sections were washed three times with PBS and mounted with mounting medium (DAKO). All images were captured under 10 or 40 × magnification using an Olympus BX-53 and confocal laser scanning microscope (Olympus FV3000). For the quantification of AQP4-, GFAP-, Iba1-, and MPO-positive areas, images of sagittal sections were taken and analyzed using ImageJ software (NIH). The region of interest (ROI) was set as 250 µm^2^ for 3 sites: centered at the lesion epicenter, 300 µm rostral to the lesion epicenter (eyeball side), and 300 µm caudal to the lesion epicenter (optic chiasma side). For each ROI, the average IHC-positive area/total area (%) from three sequential sections was determined. Then, the average of the values from three ROIs was defined as the IHC-positive area (%) of the tested animal. For the quantification of Myelin-positive areas, images of coronal sections at 1 mm and 4 mm caudal to the injection site were taken and analyzed using ImageJ software (NIH). For each site (1 mm and 4 mm), the average value of FluoroMyelin-positive area/total area (%) from three sequential sections was determined.

### Quantification of axons

Quantification of retinal ganglion cell (RGC) axons was performed according to a previously reported method [22] with minor modifications. The optic nerve samples were embedded in Tissue-Tek O.C.T. compound, then cut into 14-µm-thick coronal sections using a cryostat and placed on MAS-coated glass slides. The sections were washed three times with PBS, then incubated with FluoroMyelin™ Red and DAPI for 1 h at room temperature in the dark. The sections were washed three times with PBS and mounted with mounting medium (DAKO). Images of coronal optic nerve sections were taken under 40 × magnification using a confocal microscope (Olympus FV3000). The total number of CTB-488 positive particles at 1 mm and 4 mm caudal to the injection site were quantified using ImageJ software (NIH). For each site (1 mm and 4 mm), the average value from three sequential sections was calculated and used as the number of axons of the animal.

### Behavioral test

Quantification of the extent of pupillary light reflexes was performed as previously described [16]. Briefly, the rats were dark-adapted for 1 h before the test. The rats were then placed and fixed in a light-shielded box under weak anesthesia with isoflurane (1.5%). The pupil on the diseased side was recorded using a video camera in infrared mode. The pupil was imaged for 30 s in the dark, and light illumination was applied for 30 s thereafter. The pupil constriction rate (%) was calculated using the pupil diameter before and 3 s after illumination. Then, the light was turned off, and after 30 s, the eye on the opposite side was illuminated to assess the indirect light reflex. All rats included in this study showed prompt miosis in the eye on the diseased side, indicating that the function of the oculomotor nerve remained intact after the experimental procedure.

### Statistics

All data are presented as the mean ± SEM. Statistical comparisons were performed using Student’s *t* test and one-way and two-way analysis of variance (ANOVA), followed by Bonferroni tests. Spearman's correlation coefficient test was used to assess the correlation between the number of CTB-488 positive axons and the behavioral test of pupillary light reflex. Statistical significance was set at P < 0.05. GraphPad Prism 7 (GraphPad Software) was used for statistical analyses.

## Results

### Direct injection of high-affinity anti-AQP4 monoclonal antibody into the optic nerve induced extensive loss of AQP4, GFAP, and immune cell infiltration

To establish an NMO model of ON, we directly injected a high-affinity anti-AQP4 monoclonal antibody (E5415A) into the optic nerve. First, the expression of AQP4 by immunohistochemistry was tested (Fig. 1A). Two days after the E5415A injection, extensive loss of AQP4 was observed around the injection site (Fig. 1B). In contrast, the control-IgG injection showed no apparent change in AQP4 immunoreactivity. Next, we performed immunohistochemistry to check for the loss of GFAP and infiltration of immune cells, which are typically observed in NMO lesions. As expected, we observed extensive loss of immunoreactivity for GFAP 2 days after the E5415A injection (Fig. 1C). In contrast, the control-IgG injection induced no apparent changes in AQP4 and GFAP expression. Thereafter, the infiltration/activation of immune cells was investigated 2 days after E5415A injection and found that a number of Iba1-positive cells accumulated at the lesion site. Morphologically, these Iba1-positive cells were large and rounded cells without processes (Fig. 1F). Hence, they were considered infiltrating macrophages and partially activated microglial cells. In addition, MPO-positive neutrophils infiltrated the lesion site. Four days after the E5415A injection, the accumulation of Iba1-positive cells was exacerbated, whereas MPO-positive cells were rarely observed (Fig. 1G).

These results indicate that E5415A injection into the optic nerve can induce a prompt and extensive loss of AQP4/GFAP and subsequent infiltration of macrophages and neutrophils.

### Local injection of AQP4-IgG induced axonal injury in the optic nerve

To assess whether optic nerve axons were affected by E5415A injection, we performed a neural-tracing study. RGC axons were labeled with fluorescent-labeled CTB (Alexa Fluor 488 conjugated CTB) tracer by intravitreal injection [21]. After confirming that the RGC axons were effectively visualized 2 days after CTB-488 injection, we analyzed the extent of optic nerve damage 7 days after E5415A injection (Fig. 2A). As shown in Fig. 2B, most CTB-labeled RGC axons disappeared at the injection site, and only a few fibers crossed the lesion toward the optic chiasm. For the quantitative analysis, the number of CTB-labeled fibers in the coronally sectioned optic nerve at 1 and 4 mm caudal to the injection site was counted (Fig. 2C). As shown in Fig. 2D, E) we confirmed that AQP4-IgG induced severe axonal degeneration. Because the optic nerve injection procedure is invasive, Control-IgG injection was shown to induce moderate axonal loss (Intact group vs. Control-IgG group); nonetheless, the impact of E5415A injection was more prominent (Control-IgG group vs. AQP4-IgG group), indicating that E5415A-induced pathological reaction is highly detrimental to RGC axons. We also examined the extent of demyelination (Fig. 2F), and revealed that the FluoroMyelin-positive area around the E5415A-injected site was decreased, suggesting that focal demyelination had occurred. Taken together, E5415A-injected rats exhibited NMO-like pathological manifestations of ON, including the loss of AQP4/GFAP immunoreactivity, macrophage and neutrophil infiltration, demyelination, and axonal degeneration.

### AQP4-IgG-induced ON rats exhibited severe visual dysfunction

Given that the E5415A injection caused degeneration of RGC axons, we performed a functional assessment using the pupillary light reflex (Fig. 3A). To measure the light-induced constriction rate of the pupil, rats were dark-adapted, and dilated pupils on the tested side were recorded (dilated pupil diameter). Light stimulation was then applied, and the constricted pupil diameter was measured. In the control-IgG-injected rats, pupillary constriction was promptly induced by light stimuli, and the percentage of constriction was approximately 80% (Fig. 3B, C). In contrast, the pupillary light reflex was severely impaired (< 20%) in E5415A-injected rats, and this impairment persisted throughout the testing period (until 21 days after E5415A injection). After behavioral testing, rats received intravitreal CTB injection (at day 22) and the number of CTB-labeled RGC axons was analyzed (Fig. 3A). E5415A-induced axonal loss was still apparent in the later timepoint, (Fig. 3D). Moreover, the number of CTB-labeled RGC axons was positively correlated with visual dysfunction (Fig. 3E). These results indicated that E5415A-induced ON exhibited severe functional deficits.

### Minocycline ameliorated axonal degeneration and functional outcome in AQP4-IgG-induced ON rats

Based on the results of the immunohistochemical experiments (Fig. 1), we hypothesized that infiltrating macrophages and activated microglial cells are critical drivers of the exacerbation of E5415A-induced pathology. Therefore, we investigated whether minocycline, a potent inhibitor of macrophage/microglial activation [23], has a therapeutic effect on E5415A-induced ON.

First, we assessed the effects of minocycline on immune cell infiltration 2 days after E5415A injection (Fig. 4A) and confirmed that the Iba1-positive area was reduced by minocycline treatment (Fig. 4B, C). In contrast, minocycline administration had no significant effect on MPO-positive cell infiltration (Fig. 4D, E).

We investigated whether minocycline could ameliorate axonal degeneration and demyelination in our model. E5415A-induced ON rats received daily intraperitoneal injections of minocycline/vehicle, and on day 7, RGC axons were visualized and analyzed (Fig. 4F). As shown in Fig. 4G–M, both axonal degeneration and demyelination around the lesion site were significantly attenuated by minocycline treatment. Next, we investigated the functional outcome of minocycline treatment (Fig. 4N–Q). As expected, the light reflex impairment was significantly attenuated by minocycline administration. Further, the number of CTB-labeled RGC axons was positively correlated with visual dysfunction (Fig. 4Q).

## Discussion

In this study, we established an experimental animal model of NMO that exhibited severe ON with extensive loss of AQP4, axonal degeneration, and functional impairment. In addition, using this model, we showed that minocycline treatment improved the outcome of AQP4-IgG-induced ON.

Several direct-injection models have been reported as experimental models of NMO-IgG-related ON [14–16]. Although the loss of AQP4 around the injection site was confirmed, the pathological manifestation was mild. Moreover, functional assessment was not performed, except in one study by Zhang et al. [16]. In this report, the authors excellently demonstrated that ON rats that received a direct injection of seropositive patient-derived serum exhibited a decrease in visual evoked potential and impairment of pupillary light reflex. One of the main advantages of using patient-derived sera is their clinical relevance, as they may contain a variety of IgGs other than AQP4-IgG and soluble factors that might be involved in the pathophysiology of NMO. However, it is well-known that patient-derived sera or IgGs vary widely in their ability to generate NMO-like pathology [24–26], making it difficult for researchers to perform reproducible experiments. In addition, the potential differences in the affinity of NMO-IgG for AQP4 among species and the difference in immune reaction against pathogenic IgG derived from other species affect the development of the pathology, leading to limited exacerbation of the pathological/functional manifestations [27]. To overcome this, we used high-affinity anti-AQP4 antibodies, which were generated against rodent AQP4 (E5415A). As expected, our ON model rats demonstrated extensive loss of AQP4 and GFAP in the optic nerve, with massive infiltration of immune cells. Therefore, our strategy offers a useful and reproducible experimental model to address the pathological mechanism of AQP4-IgG-related NMO-like lesions in the optic nerve.

One of the characteristics of AQP4-associated ON is that axonal damage is more pronounced than demyelination, a major difference from MS. This is considered to be the cause of the worse visual outcomes of NMO-associated ON compared to MS-associated ON [28]. Therefore, elucidating the underlying mechanism of severe axonal damage in AQP4-associated ON is critical for establishing effective drugs to improve the visual outcomes of patients with NMO. In this study, we investigated the extent of axonal damage in our model and revealed that the number of RGC axons was reduced to approximately 25% by E5415A injection compared to control-IgG-injected rats. Consistent with this result, the rats showed severe visual dysfunction. To our knowledge, our model had the most severe AQP4-associated ON among the experimental model that enabled the elucidation of the pathophysiology of axonal injury in NMO.

The detailed mechanism of axonal degeneration in NMO has not yet been fully elucidated. Based on pathological studies of patients with NMO, the infiltrating immune cells found in the lesion sites were leukocytes, such as neutrophils, macrophages, and eosinophils [29]. Although complement-dependent cytotoxicity is considered a primary event in NMO pathology [30], the importance of antibody-dependent cellular cytotoxicity mechanism in the expansion of lesions and disease progression has become evident [31, 32]. Using a spinal cord culture system, Zhang et al. demonstrated that neutrophils and macrophages potentiated lesions induced by NMO-IgG [24]. In another study, Saadoun et al. tested the role of neutrophils in a mouse NMO model produced by intracerebral injection of NMO patient-derived IgG. They revealed that the pharmacological depletion of neutrophils ameliorates NMO pathology [33]. These results highlight the role of neutrophils and macrophages in NMO lesions, raising the possibility that these cells may directly induce neuronal damage [32]. Thus, they can be a future therapeutic target. In this study, we revealed that a large number of neutrophils and macrophages/microglia accumulated within the lesion site during the acute phase, suggesting their involvement in the expansion of NMO lesions. Particularly, macrophages/microglia accumulation was prominent 2 days after AQP4-IgG injection and further increased toward the fourth day. These observations prompted us to test the possible pharmacological inhibition of macrophages/microglia activation.

For this purpose, we treated NMO rats with minocycline, a well-known modulator of macrophages/microglia reactivity [34, 35]. We demonstrated that the accumulation of Iba1-positive cells at the lesion site was reduced by minocycline treatment, confirming that this treatment is also effective in inhibiting the activation of macrophages/microglia in AQP4-associated ON. Then, we investigated the outcome of minocycline treatment and observed that it greatly attenuated the extent of axonal injury and functional impairment induced by AQP4-IgG. Considering the underlying mechanism of action of minocycline, various detrimental roles of macrophages/microglia have shown to be ameliorated by minocycline treatment [36]. Several studies have reported that minocycline exerts anti-inflammatory properties by inhibiting activation and proliferation of macrophages/microglia through inhibition of the p38 mitogen-activated protein kinase (p38 MAPK) pathway [37, 38]. In addition, minocycline has been shown to reduce the production of inflammatory cytokines in macrophages/microglia by inhibiting the nuclear translocation of the nuclear factor-κB signal [23, 38], which is suggested to be involved in axonal degeneration [39]. Minocycline has also been shown to reduce the pathological production of matrix metalloproteinases [40], which play a destructive role in CNS integrity/homeostasis. In another study, minocycline was suggested to be neuroprotective, partially through a caspase-1-dependent mechanism [41]. Therefore, minocycline may serve as a multifunctional therapeutic agent against neuronal injury [42]. Further studies are needed to uncover the detailed therapeutic mechanisms of minocycline in AQP4-associated ON.

This study has some limitations. First, although it has been strongly suggested that minocycline exerts neuroprotective effects by inhibiting the activation of macrophages/microglia, it has a non-specific effect on other cells, including T cells and astrocytes [43, 44]. To further determine the detailed mechanism, future investigations using genetically engineered animals that enable us to conduct cell-type-specific observation and manipulation are needed. Second, we used a direct-injection technique to induce NMO lesions in the optic nerves. Although our strategy had the great advantage of inducing severe AQP4-associated lesions, it made it difficult to reproduce perivascular lesions seen in patients with NMO. In addition, it also makes it difficult to elucidate the pathological mechanism of vasocentric AQP4-IgG leakage into the optic nerve parenchyma. To overcome this limitation, the establishment of more clinically relevant experimental models is required.

## Conclusions

We established a new AQP4-associated ON model that reproduces the histological characteristics of ON in NMO patients, including loss of AQP4 and GFAP, immune cell infiltration, extensive damage of the RGC axons, and impairment of visual function, using a high-affinity anti-AQP4 monoclonal antibody. Furthermore, we revealed that minocycline treatment effectively ameliorated the anatomical and functional outcomes of AQP4-IgG-induced ON, highlighting the usefulness of our model for evaluating potential therapeutic drugs for ON in NMO.

## Supplementary Information

**Additional file 1: Fig. S1. **Pathological changes after direct injection of high-affinity anti-aquaporin-4 (AQP4) monoclonal antibody into the optic nerve. Representative images of AQP4, GFAP, Iba1, and MPO staining of sagittal optic nerve sections obtained from Intact and AQP4-IgG-injected animals at day 1, day 2, day 4, day 7, and day 24 after injection. Scale bar: 500 μm.

## Data Availability

The data sets supporting the findings of this study are available from the corresponding author upon reasonable request.

## Abbreviations

ON: Optic neuritis
AQP4: Aquaporin-4
NMO: Neuromyelitis optica
AQP4-IgG: Anti-AQP4-antibody
GFAP: Glial fibrillary acidic protein
MS: Multiple sclerosis
EAE: Experimental autoimmune encephalomyelitis
PBS: Phosphate-buffered saline
CTB: Cholera toxin subunit B
RGC: Retinal ganglion cell
MPO: Myeloperoxidase
CNS: Central nervous system
ROI: Region of Interest
