# Supplementary figures and images for: A novel aquaporin-4-associated optic neuritis rat model with severe pathological and functional manifestations
Source: J Neuroinflammation. 2022 Oct 27;19:263. doi: 10.1186/s12974-022-02623-7 (PMC9615200; doi:10.1186/s12974-022-02623-7)

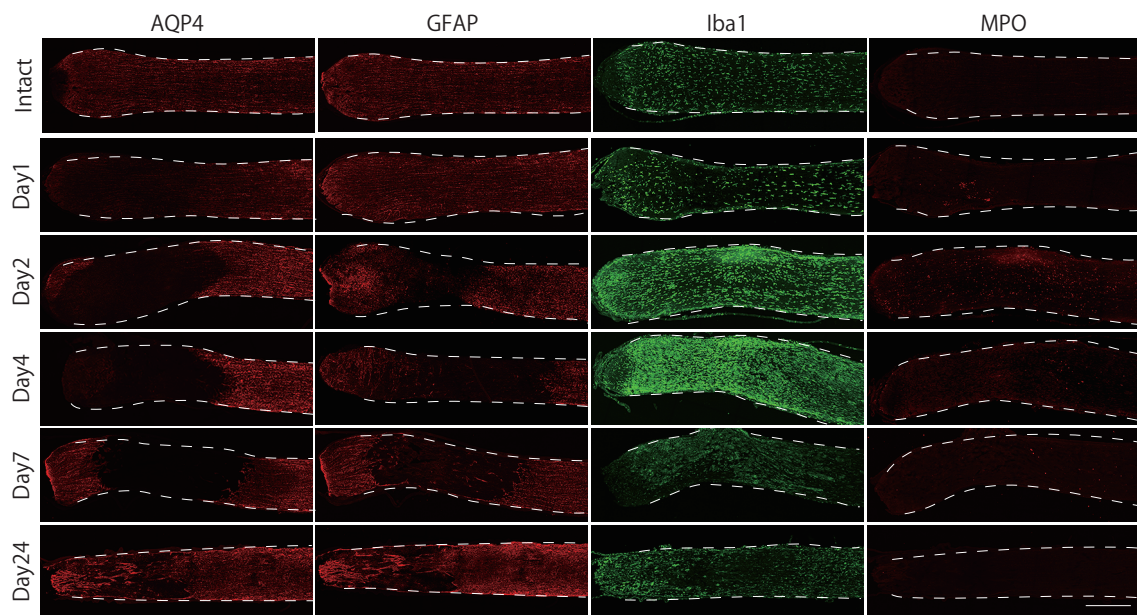

Supplement: Supplementary file 1 — Additional file 1: Fig. S1. Pathological changes after direct injection of high-affinity anti-aquaporin-4 (AQP4) monoclonal antibody into the optic nerve. Representative images of AQP4, GFAP, Iba1, and MPO staining of sagittal optic nerve sections obtained from Intact and AQP4-IgG-injected animals at day 1, day 2, day 4, day 7, and day 24 after injection. Scale bar: 500 μm. [file 12974_2022_2623_MOESM1_ESM.pdf]
